# Use of Synthetic Single-Stranded Oligonucleotides as Artificial Test Soiling for Validation of Surgical Instrument Cleaning Processes

**DOI:** 10.1155/2014/632127

**Published:** 2014-02-03

**Authors:** Nadja Wilhelm, Nadja Perle, Robert Simmoteit, Christian Schlensak, Hans P. Wendel, Meltem Avci-Adali

**Affiliations:** ^1^Department of Thoracic, Cardiac, and Vascular Surgery, University Hospital Tuebingen, Calwerstraße 7/1, 72076 Tuebingen, Germany; ^2^3mach GmbH, Ringstraße 11, 72414 Rangendingen, Germany

## Abstract

Surgical instruments are often strongly contaminated with patients' blood and tissues, possibly containing pathogens. The reuse of contaminated instruments without adequate cleaning and sterilization can cause postoperative inflammation and the transmission of infectious diseases from one patient to another. Thus, based on the stringent sterility requirements, the development of highly efficient, validated cleaning processes is necessary. Here, we use for the first time synthetic single-stranded DNA (ssDNA_ODN), which does not appear in nature, as a test soiling to evaluate the cleaning efficiency of routine washing processes. Stainless steel test objects were coated with a certain amount of ssDNA_ODN. After cleaning, the amount of residual ssDNA_ODN on the test objects was determined using quantitative real-time PCR. The established method is highly specific and sensitive, with a detection limit of 20 fg, and enables the determination of the cleaning efficiency of medical cleaning processes under different conditions to obtain optimal settings for the effective cleaning and sterilization of instruments. The use of this highly sensitive method for the validation of cleaning processes can prevent, to a significant extent, the insufficient cleaning of surgical instruments and thus the transmission of pathogens to patients.

## 1. Introduction

The complexity of some surgical instruments and their inaccessibility during routine cleaning processes hamper the residue-free removal of contaminants from the surface of instruments. Thus, the use of these inadequately cleaned instruments can lead to postoperative sepsis and the transmission of infectious diseases, such as Creutzfeldt-Jakob disease (CJD) [1–3]. Therefore, the evaluation of cleaning efficiency plays a very important role in the validation of medical cleaning and disinfection machines. Certain disinfection and sterilization of medical instruments can only be achieved with previous efficient cleaning. Residues such as tissue or blood can reduce the effectiveness of both disinfection and sterilization. Microorganisms can be reduced to a desired level faster when initial colony counts are lower.

Cleaning involves various methods, such as washing with water, with mechanical forces (i.e., with flushing pressure or with ultrasound), enzymatically, or chemically (i.e., with alkalis and oxidants or acidified cleaner). Test objects for cleaning validation can be contaminated with an artificial test soil by brushing, inserting, or pipetting a certain amount of contamination. Previous studies have used different test soils, such as citrated blood, mixtures of proteins, egg yolk, or semolina pudding, to evaluate the cleaning efficiency of medical cleaning and disinfection machines. The goal of the cleaning is to obtain an optically clean instrument in which the pathogen numbers are reduced. Residual contaminants on test objects can be determined qualitatively (e.g., by the ninhydrin or peroxidase method), semiquantitatively (e.g., by the biuret/BCA (bicinchoninic acid) method), or quantitatively (e.g., by ATP (adenosine triphosphate) determination or the modified OPA (ortho-phthaldialdehyde) method).

**Figure 1:**
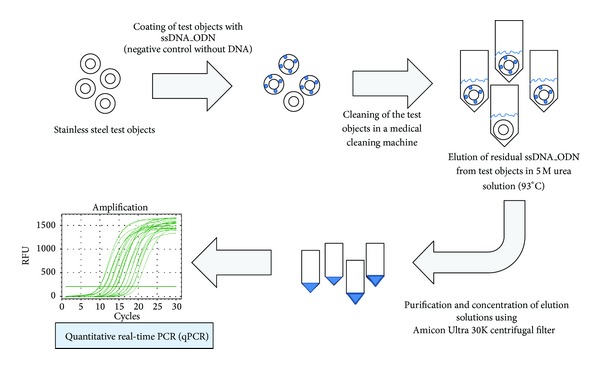
Overview of the quantification of the residual ssDNA_ODN amount on test objects after cleaning in a medical cleaning machine. Stainless steel test objects are coated with a defined amount of ssDNA_ODN and cleaned in a medical cleaning machine. Using 5 M urea solution at 93°C, residual ssDNA_ODN is eluted from the surface of test objects. Eluates are purified and concentrated using Amicon Ultra 30K centrifugal filters. The ssDNA_ODN amount is quantified using quantitative real-time PCR (qPCR).

Every microorganism contains genetic information coded in DNA or RNA. DNA is a robust biological material that can survive unscathed for tens of thousands of years, as evidenced by the isolation of genetic material from archaeological finds of bones of long-extinct animals. Previous studies have generally validated the cleaning of medical instruments by the determination of residual proteins. However, residual viral DNA on medical instruments has not been a focus of study. Zhao and colleagues demonstrated the formation of complete viruses after the injection of naked human T-cell leukemia viral DNA into the bloodstream of rabbits [4]. In other studies, it was found that after feeding mice with foreign phage M13mp18 DNA, the DNA was able to reach white blood cells, spleen cells, and liver cells via the intestinal wall mucosa and was incorporated into the mouse cell genome [5, 6]. Moreover, when M13 DNA is fed to pregnant mice, test DNA can be detected in some cells of the fetuses and of newborn animals, which suggests that the DNA is transferred through the placenta [7]. Here, we applied for the first time a synthetic single-stranded DNA (ssDNA) library, which is commonly used as starting material for the selection of DNA aptamers, as a new artificial test soiling on test objects to evaluate the cleaning efficiency of medical cleaning machines. Residual amounts of ssDNA can be ultrasensitively detected by the established quantitative real-time PCR (qPCR) after the cleaning process (Figure 1).

## 2. Materials and Methods

### 2.1. Oligonucleotides

An ssDNA oligonucleotide library (ssDNA_ODN) containing a randomized sequence region of 49 nucleotides and two fixed primer hybridization regions, 5′-GCCTGTTGTGAGCCTCCTAAC-N49-CATGCTTATTCTTGTCTCCC-3′, was used for qPCR analyses. For the amplification of ssDNA_ODN, qPCR reaction mixtures contained the forward primer 5′-GCC TGT TGT GAG CCT CCT AAC-3′ and the reverse primer 5′-GGG AGA CAA GAA TAA GCA TG-3′. All oligonucleotides were ordered HPLC purified from Ella Biotech GmbH (Martinsried, Germany).

### 2.2. Quantitative Real-Time PCR (qPCR) Analyses

For the detection and quantification of ssDNA_ODN in solutions, qPCR was performed using iQ SYBR Green Supermix (Bio-Rad Laboratories, Munich, Germany). All qPCR reactions were performed in 96-well plates. Each qPCR reaction mix was prepared by adding 10 *μ*L ssDNA_ODN solution to 50 *μ*L master mix, containing 30 *μ*L iQ SYBR Green Supermix and 360 nM forward and reverse primer. The qPCR was performed using an iCycler PCR machine (Bio-Rad Laboratories, Munich, Germany). PCR cycling conditions consisted of an initial hot start at 95°C for 3 minutes to activate the hot-start iTaq DNA polymerase and to denature the template DNA, followed by 30 cycles of denaturing at 95°C for 45 seconds, annealing at 58°C for 20 seconds, and elongation at 72°C for 20 seconds. An additional final elongation step was performed at 72°C for 5 minutes. At the end of the amplification process, a melt-curve analysis was performed consisting of 50 melt cycles, beginning at 70°C with increments of 0.5°C per cycle to control the specific length of the amplified ssDNA. After the amplification, the used ssDNA_ODN revealed a single melt curve with a maximum peak at 83°C.

Serial dilutions from 5 to 0.02 pg ssDNA_ODN and a negative control without template were included in each run. Each sample was tested in triplicate with, respectively, 15 *μ*L sample volume. All qPCR data were collected and analyzed using iCycler iQ Multicolor Real-Time PCR Detection System Software Version 3.1 (Bio-Rad Laboratories, Munich, Germany). Using the standard curve of the serial dilutions of ssDNA_ODN, the unknown quantities in the samples were calculated. For an exact determination of the amount of ssDNA_ODN in the solutions, samples were used in the correct dilution to measure within the linear range of the standard curve.

### 2.3. Occurrence of the Used ssDNA Library in Blood of Various Species

To ensure that the used ssDNA_ODN specific primers cannot amplify parts of the genomic DNA (gDNA) material, which is present in the residual blood impurities on surgical instruments, qPCR experiments were performed with different gDNA isolated from peripheral blood of different species. For this purpose, 100 ng of isolated gDNA was added to the master mix of qPCR reactions containing ssDNA_ODN specific primers. Furthermore, spiked probes that contained additionally to the gDNA known amounts of ssDNA_ODN (5 or 2.5 pg) were used as a positive control to determine the influence of the gDNA on ssDNA_ODN detection.

gDNA was isolated from the peripheral blood cells of humans (*n* = 3), pigs (*n* = 3), guinea pigs (*n* = 3), rats (*n* = 7), mice (*n* = 20), and chicks (*n* = 8). Because of the small amount of blood per rat, mouse, and chicken, blood was collected from each animal species and pooled for the isolation of cells. In contrast, the isolation of cells from human, porcine, and guinea pig blood was performed separately. Peripheral blood was collected using 1.6 mg EDTA/mL blood. To eliminate erythrocytes from the collected blood samples, whole blood was incubated in a fivefold volume of erythrocyte lysis buffer consisting of 0.15 M ammonium chloride, 10 mM potassium hydrogen carbonate, and 0.1 mM Na_2_-EDTA-2H_2_O for 15 minutes on ice and then centrifuged at 400 g for 10 min at 4°C. The supernatant was discarded, and the pellet was resuspended again in erythrocyte lysis buffer. After incubation on ice for another 5 minutes, the suspension was centrifuged at 400 g for 10 min at 4°C. This process was repeated 3-4 times. Subsequently, gDNA was isolated using the DNeasy Blood & Tissue Kit (Qiagen, Hilden, Germany) according to the manufacturer's instructions. The concentration of the isolated gDNA was determined using a spectrophotometer (ScanDrop, Analytik Jena, Jena, Germany).

### 2.4. Coating of ssDNA_ODN on Stainless Steel Screw Nuts

Stainless steel screw nuts (M5x5, steel grade 1.4301 (X5CrNi18-10), Larus Instrumente GmbH, Tuttlingen, Germany) for clinical surgery were used as test objects for the immobilization of ssDNA_ODN. These test objects were selected for experiments to mimic the adhesion of genomic material on stainless steel surgical instruments as used in the clinical praxis. Furthermore, ssDNA_ODN was applied to the surface of screw thread to imitate a location that would be difficult to access for cleaning, as in the case of many surgical instruments. The test objects were coated with 5 *μ*L ssDNA_ODN solution, which contained 5 *μ*g of ssDNA_ODN, and dried for 2 h at RT under the PCR workstation (Peqlab Biotechnologie GmbH, Erlangen, Germany).

### 2.5. Purification and Concentration of ssDNA_ODN from Eluates

To determine the residual amount of ssDNA_ODN on test objects after the cleaning process, the ssDNA_ODN was eluted using nuclease-free water or 5 M urea solution from the surface of these objects. High ion concentrations or chemicals in the eluate can affect qPCR. Therefore, ssDNA_ODN in the eluates was concentrated and purified using Amicon Ultra 0.5 mL 30K centrifugal filters (Millipore GmbH, Schwalbach/Ts., Germany) according to the manufacturer's instructions to prevent an effect on qPCR. Urea concentrations less than 8 M are compatible with the filter. The used ssDNA_ODN had a length of 90 nucleotides and an approximate molecular weight of 29.7 kDa. Therefore, Amicon Ultra filters with a 30 kDa molecular weight cut-off were selected to ensure that the ssDNA_ODN remained in the collection tube. During centrifugation, molecules ≥ 30 kDa cannot flow through the filter, and they are collected, whereas all the smaller molecules are eluted.

To examine the efficiency of the purification method, 500 *μ*L of water or 5 M urea solution containing 10 *μ*g ssDNA_ODN for gel electrophoresis experiments was filtered using Amicon Ultra 0.5 mL 30K centrifugal filters. Thereby, a concentrate and flow-through were obtained. Both the concentrate and flow-through were filled up to 500 *μ*L with nuclease-free water. For gel electrophoresis, 300 ng of the ssDNA_ODN as a positive control and 15 *μ*L of concentrate and flow-through were run on 10% denaturing urea-polyacrylamide gel. The gel was stained with GelRed (Biotium Inc., Hayward, USA) and documented using the gel documentation system (Gel Doc XR, Bio-Rad Laboratories GmbH, Munich, Germany).

### 2.6. Establishment of the Elution of ssDNA_ODN from Test Objects

To establish an efficient method for elution of the ssDNA_ODN from the surfaces of test objects, ssDNA_ODN coated test objects were incubated three times each for 10 minutes with 250 *μ*L of nuclease-free water (*n* = 3) or 5 M urea solution (*n* = 3) at 93°C to determine the best elution method. Every 10 minutes, test objects were taken out of the elution solution and transferred to the next fresh elution solution. Positive controls were prepared by adding 5 *μ*g ssDNA_ODN in 250 *μ*L water (*n* = 3) or in 5 M urea solution (*n* = 3). Then, they were treated in accordance with the eluent for the test objects. After elution, the ssDNA_ODN in all elution solutions and positive controls was concentrated and purified using Amicon Ultra 0.5 mL 30K centrifugal filters. All concentrates with purified ssDNA_ODN were filled up to 500 *μ*L with nuclease-free water. For gel electrophoresis, 15 *μ*L of concentrates was run on 10% denaturing urea-polyacrylamide gel. The gel was stained with GelRed (Biotium Inc., Hayward, USA) and documented using the gel documentation system (Gel Doc XR, Bio-Rad Laboratories GmbH, Munich, Germany). The concentrates for qPCR experiments were diluted 1 : 10,000 in nuclease-free water, and the amount of ssDNA_ODN was quantified using qPCR. Measurements were repeated three times.

### 2.7. Influence of Possible Residual Chemicals in Concentrated ssDNA_ODN Eluates on qPCR

A possible influence of the used 5 M urea elution solution on qPCR was examined. Therefore, 500 *μ*L of 5 M urea solution (*n* = 3) without ssDNA_ODN was filtered using Amicon Ultra 30K centrifugal filters according to the manufacturer's instructions. The concentrated samples were filled up to 500 *μ*L with nuclease-free water. The influence of concentrates on qPCR was tested by adding 10 *μ*L of each sample to 40 *μ*L of master mix and spiking this mixture with 10 *μ*L of ssDNA_ODN solution containing defined amounts of ssDNA_ODN from 5 pg to 0.02 pg, as used for the standard curve. Detected Ct (cycle threshold) values of samples were then compared with the Ct values of the standard to determine possible changes.

### 2.8. Cleaning of Test Objects in a Laboratory Cleaning Machine and Determination of Residual ssDNA_ODN Amounts on Test Objects

To verify the suitability of the used test method for the cleaning validation of medical cleaning machines, cleaning experiments were performed using a laboratory cleaning machine (Mielabor G7783 Multitronic, Miele, Gütersloh, Germany) with different cleaning temperatures and periods. Three test objects (*n* = 3) with 5 *μ*g ssDNA_ODN coating and one negative control without ssDNA_ODN coating were washed at 60°C, 85°C, or 93°C for 10, 20, 25, and 30 minutes. The cleaning process was performed with and without detergent (neodisher MA, Dr. Weigert, Hamburg, Germany). After every cleaning process, elution of the residual ssDNA_ODN from the test objects was performed by heating them once at 93°C for 10 minutes in 250 *μ*L of 5 M urea solution. Subsequently, the test objects were taken out of the hot solutions, and the eluates were purified and concentrated using Amicon Ultra 0.5 mL 30K centrifugal filters. Positive controls were prepared by adding 5 *μ*g ssDNA_ODN in 250 *μ*L 5 M urea solution and heating for 10 min at 93°C. They were also purified analogous to eluates from test objects using Amicon Ultra 30K filters. All concentrated samples were filled up to 500 *μ*L with nuclease-free water and used in appropriate dilutions for qPCR.

## 3. Results

### 3.1. ssDNA_ODN Detection Range of qPCR Assay

In this study, an ssDNA library (ssDNA_ODN) that contained a randomized sequence region of 49 nucleotides and two fixed primer hybridization regions was used as a test soiling. The primer regions with known sequences enabled the specific amplification of ssDNA_ODN by qPCR. For the quantitative detection of ssDNA_ODN amounts in unknown samples, a standard curve presenting Ct values as a function of log amount of ssDNA_ODN was used. Per reaction mixture, ssDNA_ODN amounts from 5 pg to 0.02 pg (Supplementary Figure 1 in supplementary material available online at http://dx.doi.org/10.1155/2014/632127) were detected sensitively and reproducibly by using the SYBR Green I-based qPCR method. The efficiency of the qPCR was 99.4%, which shows doubling of the amplicon at each cycle. The lower detection limit was 20 pg.

### 3.2. Verification of the Specificity of the Used ssDNA_ODN Primer and Absence of the Interaction with Genomic DNA of Various Species

**Figure 2:**
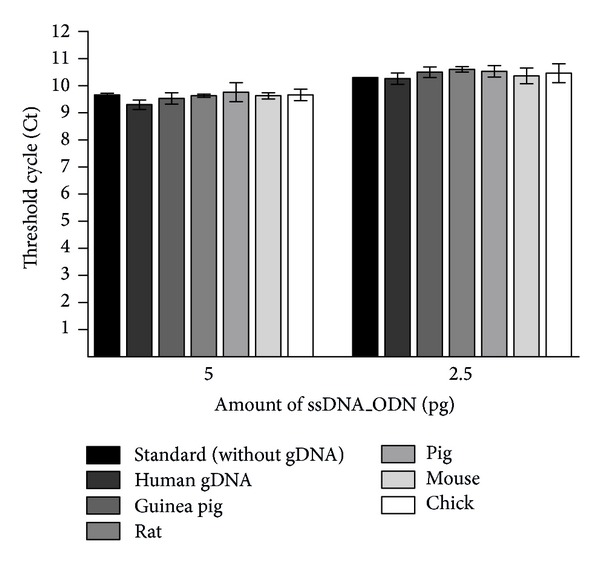
Investigation of primer binding to the genomic DNA (gDNA) of various species and influence of gDNA existence on ssDNA_ODN detection. Isolated gDNA was spiked with 2.5 and 5 pg of ssDNA_ODN, and the determined Ct values were compared with the Ct values without the addition of gDNA (called standard).

Medical instruments are often contaminated with blood. Thus, during the cleaning process, blood cells that are located on the surface of dirty instruments can be thrown by mechanical forces onto the surface of test objects containing defined amounts of ssDNA_ODN. Hence, the unfavorable impact of residual gDNA from blood cells on qPCR detection of ssDNA_ODNs has to be determined. Therefore, qPCR assays were performed with gDNA samples isolated from peripheral blood of humans, pigs, guinea pigs, rats, mice, and chicks by adding primers to the reaction mixture. Furthermore, gDNA samples were spiked with 5 or 2.5 pg ssDNA_ODN to obtain positive controls and to investigate the influence of gDNA samples on ssDNA_ODN detection (Figure 2). Without the addition of template ssDNA_ODN, the detected Ct values of samples were outside of the detection range (data not shown), which shows that the primers do not lead to the unspecific amplification of gDNA regions and that they are specific for used ssDNA_ODN. The addition of gDNA to 5 or 2.5 pg ssDNA_ODN demonstrated that the qPCR detection of ssDNA_ODN amount is not influenced by the existence of gDNA, since detected Ct values were not significantly different from Ct values of samples without gDNA.

### 3.3. Applicability of Amicon Ultra 30K Centrifugal Filters for Purification and Concentration of ssDNA_ODN from Eluates

**Figure 3:**
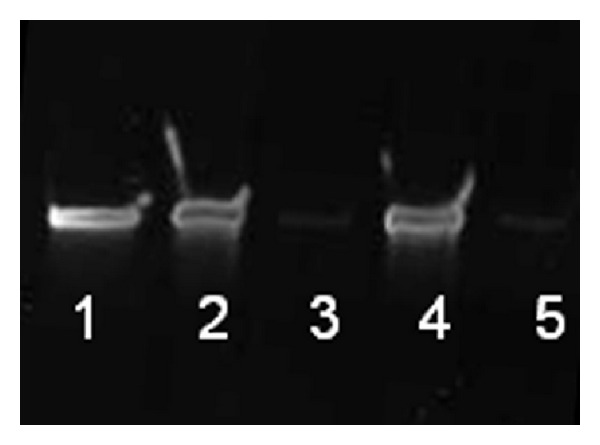
Examination of ssDNA_ODN amounts in the concentrate and flow-through after centrifugation of water or 5 M urea solutions containing 10 *μ*g ssDNA_ODN using Amicon Ultra 30K centrifugal filters. Samples of 15 *μ*L were analyzed on a 10% denaturing urea-polyacrylamide gel. Lane 1: 300 ng ssDNA_ODN (positive control), Lane 2: concentrate of water solution, Lane 3: flow-through of water solution, Lane 4: concentrate of 5 M urea solution, and Lane 5: flow-through of 5 M urea solution.

The suitability of Amicon Ultra 0.5 mL 30K centrifugal filters for the purification and concentration of ssDNA_ODN from eluates was tested by adding a defined ssDNA_ODN amount, namely, 10 *μ*g, to water or 5 M urea solution. The presence of ssDNA_ODN in the concentrate and flow-through was analyzed by gel electrophoresis. As seen in Figure 3, Lanes 2 and 4, the major amount of ssDNA_ODN was located in the concentrates. The flow-through showed very weak ssDNA_ODN bands, which indicate very small amounts of ssDNA_ODN. The ssDNA_ODN amounts in the flow-through and concentrates were also verified by qPCR (data not shown) and confirmed the results of gel electrophoresis. Both the results of gel electrophoresis and qPCR demonstrated that most of the ssDNA_ODN is located in the concentrate. Thus, concentrates were used for qPCR assays to determine residual ssDNA_ODN amounts on test objects. The use of Amicon Ultra 30K centrifugal filters simultaneously enables the elimination of molecules smaller than 30 kDa, such as salts, from the eluate. Thereby, a negative influence of the salts on the qPCR can be prevented.

### 3.4. Elution of ssDNA_ODN from Test Objects

**Figure 4:**
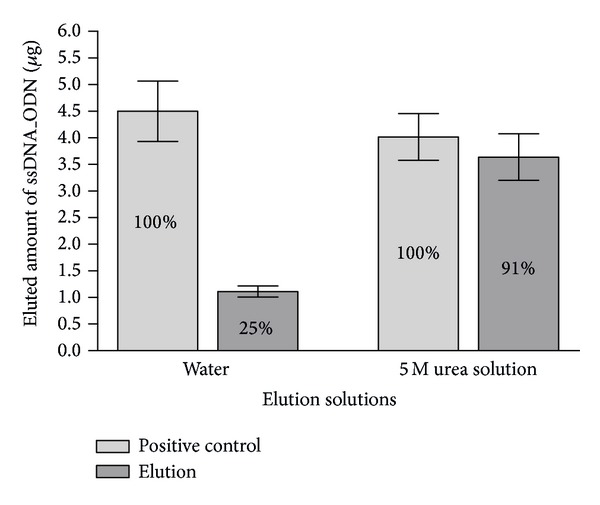
Comparison of ssDNA_ODN elution efficiency of water or 5 M urea solution at 93°C from the surface of test objects. Using qPCR, detectable amounts of ssDNA_ODN were determined after the elution of immobilized ssDNA_ODN in water or 5 M urea solution (*n* = 3). The ssDNA_ODN amount (5 *μ*g) used for coating of test objects was added to each elution solution. The detected ssDNA_ODN amounts after the Amicon Ultra 30K centrifugal filter purification and concentration were set to 100% (positive controls). The results are presented relative to the positive control as means ± SEM.

Stainless steel screw nuts were used as test objects for the immobilization of 5 *μ*g ssDNA_ODN. To find the best elution method, ssDNA_ODN coated test objects were incubated three times each for 10 minutes with nuclease-free water or 5 M urea solution at 93°C. Positive controls were prepared by adding 5 *μ*g of the used ssDNA_ODN for coating in water or in 5 M urea solution. The ssDNA_ODN in the eluates and positive controls was concentrated and purified using Amicon Ultra 0.5 mL 30K centrifugal filters. The amount was determined by qPCR. The purification and concentration of eluates using centrifugal filters were associated with an average ssDNA_ODN loss rate of 20% in 5 M urea solution samples and of 10% in water samples. Presumably, the loss occurred during the pipetting of the solution into the filters and the elution of the concentrate from the filter. Therefore, the detected total amount of ssDNA_ODN after the purification with centrifugal filter, which is named as positive control, was set to 100%. As shown in Supplementary Table 1, using 5 M urea solution at 93°C, 91% of the immobilized ssDNA_ODN was eluted from the surface of test objects in the first elution step (Figure 4), while the second elution step contained 6% of the used ssDNA_ODN. Only 0.4% of the total ssDNA_ODN was detectable in the third elution step. These results clearly demonstrate that most of the ssDNA_ODN is efficiently eluted already after the first incubation with 5 M urea solution at 93°C for 10 min. Using water as an elution solution, the first eluate also contained the most ssDNA_ODN; however, after the first elution step, only 25.6% of the immobilized ssDNA_ODN could be eluted (Figure 4), which was approximately 3.5 times lower than the elution with 5 M urea solution. Thus, for cleaning validation, the incubation of test objects once for 10 min at 93°C with 5 M urea solution is sufficient to elute the ssDNA_ODN. The denaturing polyacrylamide gel electrophoresis analyses (Supplementary Figure 2) confirmed the results of the qPCR. The main amount of ssDNA_ODN was detected in the first eluate. A very faint band was seen in the second eluate, and no band was seen in the third eluate.

### 3.5. Influence of Possible Residual Chemicals in Concentrated ssDNA_ODN Eluates on qPCR

**Figure 5:**
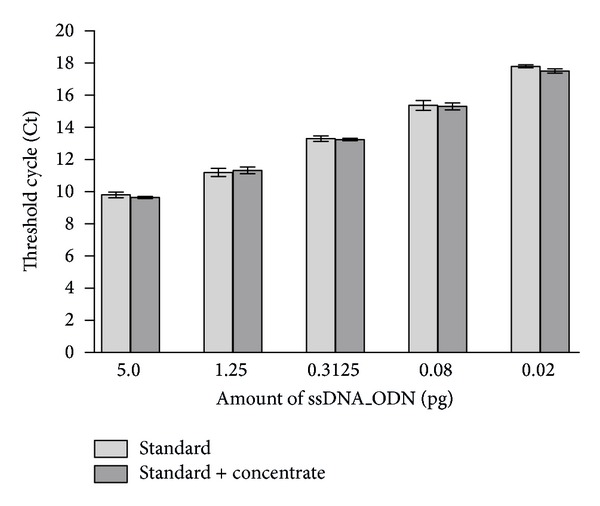
Determination of the influence of possible urea residues in the concentrated eluates on qPCR detection. Ct values of standard with addition of concentrates from 5 M urea solution were compared with the Ct values of the standard (*n* = 3). The results are presented as means ± SEM.

After the purification of the ssDNA_ODN from 5 M urea elution solution, urea residues may remain in the concentrates, which can lead to the inhibition of qPCR. It is well known that urea concentrations of ≥50 mM in PCR reactions inhibit PCR. Therefore, to examine whether residuals from the 5 M urea elution solution influence the qPCR detection of ssDNA_ODN, 5 M urea solution without ssDNA_ODN was filtered and concentrated as performed during the elution of ssDNA_ODN from the surface of test objects. The standard used for the quantification was spiked with 10 *μ*L of these concentrates. Detected Ct values of standard containing concentrate were then compared with the Ct values of the standard. As seen in Figure 5, the Ct values of standard samples containing concentrate were not significantly different from Ct values of standard without the addition of concentrate. These results verified that the concentrated 5 M urea solution does not influence the accurate detection of ssDNA_ODN by qPCR.

### 3.6. Cleaning of Test Objects in a Laboratory Cleaning Machine and Determination of Residual ssDNA_ODN Amounts on Test Objects

Cleaning of test objects was performed with or without detergent in a laboratory cleaning machine with different cleaning temperatures (60, 85, and 93°C) and washing periods (10, 20, 25, and 30 min).  Table 1 shows the detectable ratio (‰) of ssDNA_ODN from positive control. After cleaning with detergent, only minimal amounts of ssDNA_ODN were detected after 10 and 20 minutes; however, after 25 minutes, no ssDNA_ODN could be detected. In contrast, cleaning without detergent resulted in the detection of ssDNA_ODN on all test objects. The lowest ssDNA_ODN amounts were detected after the cleaning at 93°C both with and without detergent. Only after cleaning at 93°C and without detergent, 0.14*‰*  of the positive control was detected after 10 min and 0.16*‰*  of the positive control was detected after 20 min on negative controls (data not shown).

## 4. Discussion

In this study, ssDNA molecules with a length of 90 nucleotides were used as a test soiling on stainless steel test objects to evaluate the efficiency of medical cleaning processes. Using qPCR, residual amounts on test objects were quantified specifically and sensitively. The used ssDNA_ODN contained a randomized sequence region of 49 nucleotides and two fixed primer hybridization regions, which allowed the specific detection during qPCR. However, instead of randomized ssDNA molecules, a fixed sequence with a defined nucleotide sequence containing primer hybridization regions could also be used.

The ISO 15883 specifies general requirements for the validation of washer disinfectors intended to be used for the cleaning and disinfection of reusable medical devices. Acceptable protein levels on processed instruments have to be below the detection limit of 2 mg/m^2^ for the ninhydrin assay, 30–50 *μ*g for the BCA assay, or 0.003 *μ*mol for the OPA assay [8]. However, to detect lower levels of contaminants, techniques with greater quantitative sensitivity are necessary. Thus, the fluorescent microscopy technique involving the visualization of protein by SYPRO Ruby staining, which is able to detect 85 pg/mm^2^, was developed [9]. More sophisticated detection techniques include, for example, surface plasmon resonance (SPR), X-ray photoelectron spectroscopy (XPS), and time of flight secondary ion mass spectroscopy (ToF-SIMS). The protein-detection limits of these techniques are 15 pg/mm^2^, 100 pg/mm^2^, and 1 pg/mm^2^, respectively [10, 11]. However, these detection methods are too expensive and complex and therefore not easy to perform. In contrast, real-time PCR cyclers are standard equipment of molecular laboratories in hospitals. Thus, our established method allows the easy, rapid, and ultrasensitive detection of 20 fg ssDNA_ODN on test objects by qPCR.

The presence of gDNA in qPCR reaction mixtures had no influence on the accurate quantification of ssDNA_ODN amount. Thereby, the uniqueness of the primers for the detection of ssDNA_ODN on test objects was demonstrated. There was no interaction with the genomic material of humans, pigs, guinea pigs, rats, mice, or chicks. The primer regions of ssDNA_ODN, which were specifically designed by Mayer et al. for efficient and specific amplification, showed no interaction with the tested gDNAs [12]. Thus, the used ssDNA_ODN coated test objects could also be used to determine the cleaning efficiency of animal surgery cleaning machines.

To elute immobilized ssDNA_ODN from test objects, 5 M urea solution or water was used. Urea is a good hydrogen bond donor and an excellent receptor. Thus, we suppose that the disruption of hydrogen bonds and/or disruption of base stacking led to the better elution of ssDNA_ODN from the surface of stainless steel test objects than when only water was used as an elution solution [13, 14]. Additionally, the incubation of the test objects at 93°C with elution solutions could have contributed to the increased release of ssDNA_ODN from the surface into the elution solution.

Several compounds of blood or tissue samples and of DNA extraction and purification methods, such as EDTA, hemoglobin, heparin, SDS, phenol, chloroform, ethanol, or salts, can influence PCR [15–17]. Thereby, an influence can occur by direct interaction with the template DNA or the DNA polymerase, which results in failure of amplification or increased or inhibited activity of the enzyme. Betaine (1 M), DMSO (1–10%), and formamide (1–10%) are some of the well-known PCR enhancing agents. An influenced PCR can result in incorrect or false amplifications or reduced sensitivity [18]. Therefore, purification of samples before PCR is necessary to avoid false results [19]. Considering these facts, in this study, all elution samples were purified to remove all interfering molecules. Urea has a molecular weight of 60.06 Da; thus, the use of Amicon Ultra centrifugal filters with a 30 kDa molecular weight cut-off ensured the elimination of urea so that qPCR reactions were not influenced.

The detection of residual ssDNA_ODN on test objects washed for up to 20 minutes with detergent and the presence of ssDNA_ODN on all test objects washed for up to 30 minutes without detergent clearly showed the influence of detergents on the cleaning of medical instruments. Furthermore, the impact of temperature on the effectiveness of cleaning was clearly demonstrated at 93°C. Here, the detected amounts were the lowest after cleaning both with and without detergent. The detection of ssDNA_ODN on negative controls after washing at 93°C showed that during the cleaning process, the ssDNA_ODN on test objects can be thrown due to mechanical forces to other locations in the cleaning machine. This phenomenon can also happen in reality with protein or DNA contaminants on instruments. Thereby, instruments that are originally not contaminated can become contaminated during the mechanical cleaning process. This shows the importance of the validation of medical cleaning and disinfection processes. The necessary time, temperature, and rinsing agent have to be determined to ensure the use of only sterile instruments without infectious proteins and DNA for medical interventions.

## 5. Conclusions

This study demonstrated for the first time the applicability of synthetic ssDNA oligonucleotides as a test soiling on stainless steel surfaces for the validation of medical cleaning processes. The established qPCR method enables the ultrasensitive and highly specific quantification of the residual synthetic ssDNA test soiling on test objects. The use of this highly sensitive method can greatly contribute to the prevention of pathogen transfer via insufficient cleaning of medical instruments.

## Supplementary Material

Supplementary Figure 1: Representation of the dynamic range of qPCR using 5, 2.5, 1.25, 0.625, 0.3125, 0.08, and 0.02 pg ssDNA_ODN.
Supplementary Figure 2: Detection of the eluted ssDNA_ODN from test objects after three successive elutions in 5M urea solution or water using denaturing urea polyacrylamide gel electrophoresis.
Supplementary Table 1: Detection of ssDNA_ODN amounts in the three successive elutions in 5 M urea solution or water using qPCR.

## Figures and Tables

**Table 1:** Determined residual content of ssDNA_ODN (‰) from immobilized amount (5 *μ*g) on test objects after cleaning at different temperatures (60, 85, and 93°C) and times (10, 20, 25, and 30 min) with and without detergent (*n* = 3).

Cleaning temperature	Cleaning	Cleaning time			
		10 min	20 min	25 min	30 min
60°C	With detergent	17.67	17.01	n.d.	n.d.
	Without detergent	41.80	56.36	23.15	14.85

85°C	With detergent	45.34	n.d.	n.d.	n.d.
	Without detergent	11.41	34.28	2.49	9.38

93°C	With detergent	0.43	0.22	n.d.	n.d.
	Without detergent	5.31	0.36	0.19	0.14

n.d.: not detectable.
