# Supplementary material for: Use of Synthetic Single-Stranded Oligonucleotides as Artificial Test Soiling for Validation of Surgical Instrument Cleaning Processes
Source: Biomed Res Int. 2014 Feb 3;2014:632127. doi: 10.1155/2014/632127 (PMC3930025; doi:10.1155/2014/632127)
Supplement: Supplementary file 1 — Supplementary Figure 1: Representation of the dynamic range of qPCR using 5, 2.5, 1.25, 0.625, 0.3125, 0.08, and 0.02 pg ssDNA_ODN. Supplementary Figure 2: Detection of the eluted ssDNA_ODN from test objects after three successive elutions in 5M urea solution or water using denaturing urea polyacrylamide gel electrophoresis. Supplementary Table 1: Detection of ssDNA_ODN amounts in the three successive elutions in 5 M urea solution or water using qPCR. [file 632127.f1.pdf]

Supplementary information

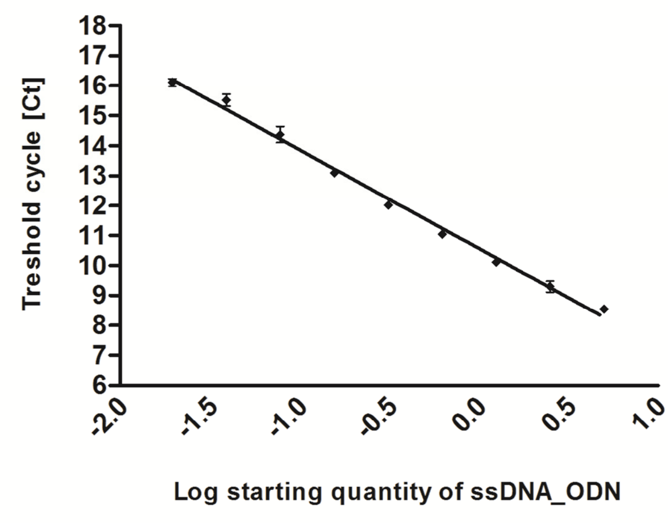

Supplementary Figure 1: Dynamic range of the quantitative real-time PCR (qPCR). The ssDNA_ODN amounts (5, 2.5, 1.25, 0.625, 0.3125, 0.08, and 0.02 pg) are plotted logarithmically. The corresponding cycle threshold (Ct) values are plotted on the y-axis.

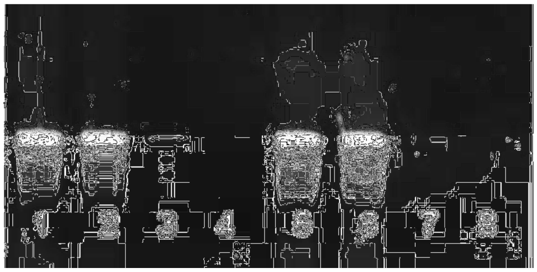

Supplementary Figure 2: Denaturing urea polyacrylamide gel electrophoresis analyses for detection of the eluted ssDNA_ODN from test objects after three successive elutions in 5 M urea solution or water. Samples of 15 µl were run on polyacrylamide urea gels. Lane 1: positive control of 5 M urea solution, Lane 2: First eluate using 5M urea solution, Lane 3: Second eluate using 5M urea solution, Lane 4: Third eluate using 5M urea solution, Lane 5: positive control of water, Lane 6: First eluate using water, 7: Second eluate using water, 8: Third eluate using water.

Supplementary Table 1: Detected ssDNA_ODN amounts by qPCR in the three successive elutions in 5 M urea solution or water (n=3).

	ssDNA_ODN amount [µg]			
	**positive control**	**1. eluate**	**2. eluate**	**3. eluate**
**Elution with 5 M urea solution**	4 ± 0.44	3.6 ± 0.44	0.24 ± 0.05	0.01 ± 0.002
**Elution with water**	4.5 ± 0.57	1.11 ± 0.11	0.001 ± 0.0002	n.d.

n.d.: not detectable
